# Damage to cardiac vasculature may be associated with breast cancer treatment-induced cardiotoxicity

**DOI:** 10.1186/s40959-021-00100-3

**Published:** 2021-04-19

**Authors:** Rebecca K. Hoffman, Bang-Jin Kim, Payal D. Shah, Joseph Carver, Bonnie Ky, Sandra Ryeom

**Affiliations:** 1 Department of Cancer Biology, Perelman School of Medicine at the University of Pennsylvania, Philadelphia, PA USA; 2 Laboratory of Innovative & Translational Nursing Research, School of Nursing at the University of Pennsylvania, Philadelphia, PA USA; 3 Division of Hematology-Oncology, Department of Medicine, Perelman School of Medicine at the University of Pennsylvania, Philadelphia, PA USA; 4 Cardio-Oncology Center of Excellence, Abramson Cancer Center, Perelman School of Medicine, University of Pennsylvania, Philadelphia, PA USA; 5 Division of Cardiovascular Medicine, Perelman School of Medicine at the University of Pennsylvania, Philadelphia, PA USA

**Keywords:** Breast cancer, Chemotherapy-induced toxicity, Doxorubicin, Cardiac vasculature, Endothelial cells

## Abstract

**Background:**

Breast cancer is the most common female cancer worldwide. Effective therapies including doxorubicin and trastuzumab have improved survival, but are associated with a substantial risk of cardiovascular disease. Mechanisms underlying cancer treatment-induced cardiotoxicity (CTC) are poorly understood and have largely focused on cardiomyocyte damage, although other cellular populations in the heart such as the cardiac endothelium, may play an important role in cardiac damage. We treated a breast tumor-bearing mouse model with doxorubicin and trastuzumab to investigate the role of the cardiac endothelium in the development of CTC.

**Methods:**

Immune compromised mice were inoculated in the 4th mammary fat pad with human breast cancer cells overexpressing HER2 (BT474). When tumors were palpable, mice were treated weekly with doxorubicin (5 mg/kg) and trastuzumab (4 mg/kg). The cardiac phenotype of mice was assessed by echocardiography and histological evaluation of the heart. Cardiac vascular damage was assayed by in vivo permeability assays and primary cultures of murine cardiac endothelial cells were used to assay doxorubicin toxicity in vitro*.*

**Results:**

The growth of BT474 breast tumors in Balb/c Nude mice was suppressed upon treatment with doxorubicin and trastuzumab. Mice treated for 4 months with doxorubicin and trastuzumab maintained body weights, but demonstrated an echocardiographic phenotype consistent with preserved left ventricular (LV) ejection fraction, decreased LV mass and increased filling pressures (E/e’). Histological staining with Masson’s trichrome and Picrosirius red showed extensive fibrosis and increased collagen deposition in the ventricular myocardium surrounding blood vessels of treated mice compared to untreated mice. Evans blue permeability assays demonstrated increased cardiac vasculature permeability while primary cardiac endothelial cells exposed to doxorubicin in vitro showed increased cell death as compared to lung or liver endothelial cells.

**Conclusions:**

An orthotopic mouse model of human breast cancer in Nude mice treated with doxorubicin and trastuzumab resulted in a cardiac vascular defect accompanied by preserved LV ejection fraction, decreased LV mass, suggesting mild diastolic dysfunction and cardiac remodeling consistent with subclinical cardiotoxicity. Our data suggest that cardiac endothelium is more sensitive to doxorubicin therapy as compared to other organ endothelium and cardiac endothelial damage may correlate with breast cancer treatment-induced cardiotoxicity.

## Background

Breast cancer is the most common female cancer worldwide, affecting over 3.3 million women in the US alone [1]. Highly effective therapies, including anthracyclines, trastuzumab (TRZ), and radiotherapy have resulted in improved cancer survival rates. However, these therapies are associated with a substantial risk of cardiovascular (CV) disease. Cardiotoxicity is a significant issue in the short-term, as cardiomyopathy and heart failure can result in treatment interruptions [2, 3], and in the long-term, as CV mortality exceeds that of cancer in survivors [4–6]. Despite numerous studies, our understanding of the cellular and molecular mechanisms underlying CV disease in this growing population of survivors remains limited.

Dose-dependent left ventricular dysfunction, cardiomyopathy, and heart failure is an established toxicity of doxorubicin (DOX) therapy [7–10]. TRZ is a humanized monoclonal antibody that disrupts ErbB2 (HER2/neu) signaling, and although its use has revolutionized the care of HER2+ breast cancer, it can result in clinically significant cardiotoxicity. The ErbB2 pathway plays a fundamental role in the maintenance of cardiac homeostasis, myocardial repair, and angiogenic signaling [11, 12]. These perturbations result in adverse cardiac and vascular remodeling and left ventricular dysfunction develops in 13% of patients receiving TRZ, and up to 27% of patients receiving combination DOX and TRZ [3, 7].

Published murine studies have suggested roles for mitochondrial damage from reactive oxygen species, metabolic ion dysregulation, and perturbations of cardiac-specific gene regulation that resulted in cardiomyocyte damage or death. However, while many studies have examined cardiotoxicity after treatment of wild-type mice with anthracyclines or radiation, most studies have not included tumors in their experimental design [13, 14]. Since studies suggest that the presence of tumor may affect CV function [15], it is important to utilize tumor-bearing mouse models to study cardiotoxicity associated with cancer treatment.

Endothelial cell (ECs) are one of the most abundant cell types in the heart and their dysfunction been shown to contribute to CV disease [16]. However the role of cardiac ECs in chemotherapy-induced CV toxicity has not been extensively investigated. ECs form the lining of blood vessels, where they help maintain vascular tone and permeability. Upon activation or damage, ECs secrete growth factors such as vascular endothelial growth factor that mobilizes endothelial progenitor cells for vascular repair, and inflammatory factors such as IL-6, TNFα, and intercellular adhesion molecule 1, initiating multiple signaling cascades promoting inflammation, production of reactive oxygen species, and platelet activation [17].

In this study, we utilized an orthotopic mouse model of breast cancer treated with DOX/TRZ to investigate the contribution of cardiac ECs to cardiovascular disease. HER2+ human breast cancer cells [18] were inoculated into the mammary fat pad of immunocompromised Nude mice and treated with DOX-TRZ at a similar dosing and interval as patients with breast cancer. Our data show adverse remodeling in the heart after 2 and 4 months of treatment, with changes in echocardiography, increased cardiac troponin-I (cTnI) and brain natriuretic peptide (BNP) levels and increased cardiac fibrosis as indicated by Masson’s trichrome and Picrosirius red staining. Our studies suggest that increased cardiac vascular permeability with decreased expression of junctional proteins in the heart may contribute to cardiac damage during DOX/TRZ treatment through direct exposure of cardiac myocytes to DOX. Further our data indicate cardiac but not lung or liver ECs are sensitive to DOX treatment in vitro

## Methods

### Animals

Female BALB/c Nude mice (C.Cg/AnNTac-*Foxn1*^*nu*^ NE9) were purchased from Taconic Biosciences at 5 weeks of age and housed in an AALAC-accredited Small Animal Barrier Facility. Female B6 nude (B6NU) mice sp./sp. (B6.Cg/NTac-*Foxn1*^*nu*^ NE10) were generated by breeding of male B6NU sp./sp. (B6.Cg/NTac-*Foxn1*^*nu*^ NE10) with female B6NU sp./wt (B6.Cg/NTac-*Foxn1*^*nu*^ NE10) mice. All animals and protocols were approved by the University of Pennsylvania IACUC review committee.

### Study design

Female Balb/c Nude mice (5–8 weeks old) were inoculated in the 4th mammary fat pad with human breast cancer cells; while control mice received sterile phosphate buffered saline (PBS) and Matrigel. Mice that developed tumors received either PBS or combination cancer treatment (5 mg/kg doxorubicin (DOX), 4 mg/kg trastuzumab (TRZ), on alternate days by intraperitoneal injection) when tumors reached 100 mm^3^. Mice that did not receive xenografts got sham injections of sterile PBS. Mice were weighed twice per week and tumors were palpated/measured twice per week. Mice were maintained on chemotherapy/sham injections for the duration of the studies. A third study included mice that were injected with PBS and Matrigel only but were given DOX/TRZ treatment for 2 months for vessel permeability studies (Table 1 for all study designs). For Study I, cumulative doses of drug per mouse in cohort 1 was DOX = 40 mg/kg and TRZ = 32 mg/kg. In Study II, cumulative doses of drug for each mouse in cohort 1 was DOX = 80 mg/kg and TRZ = 64 mg/kg. In Study III, cumulative doses of drug for each mouse in cohort 1 was DOX = 40 mg/kg and TRZ = 32 mg/kg. In cohort 3, cumulative dose of drug for each mouse was DOX = 40 mg/kg.

**Table 1:**
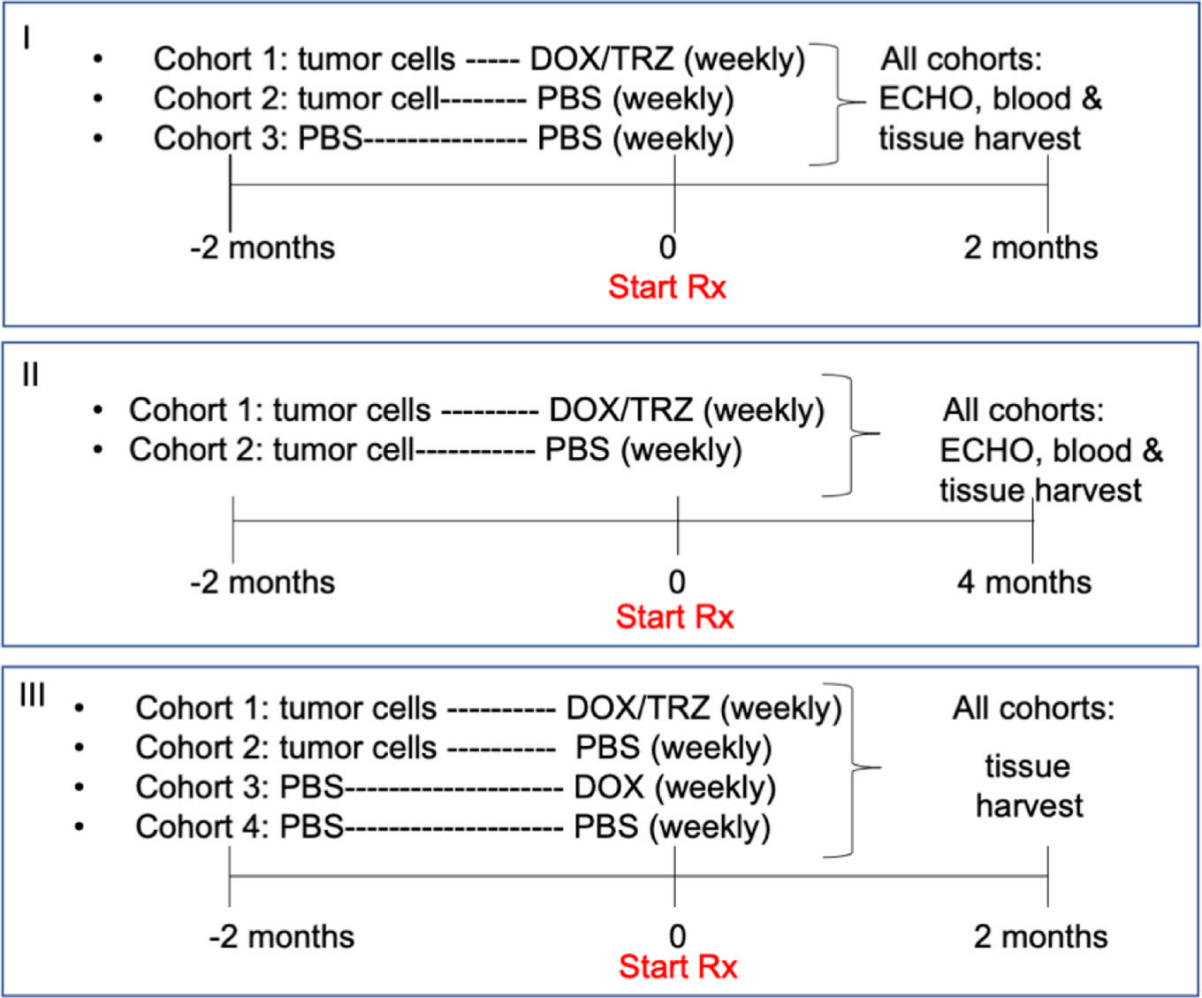
Study design

### Chemotherapy

Doxorubicin hydrochloride (DOX, Sigma-Aldrich D1515) was dissolved in sterile phosphate buffered saline (2 mg/ml), filter-sterilized, and diluted with sterile PBS to deliver 5 mg/kg in 100 μl, as an intra-peritoneal (IP) injection two times per week. Trastuzumab (TRZ, Herceptin™) was diluted with sterile PBS to deliver 4 mg/kg in 100 μl, as an IP injection once per week, on alternate days with DOX.

### Xenograft procedure

BT474 cells (ATCC HTB-20), were plated onto 0.1% gelatin-coated T-175 culture flasks and grown to 70% confluence (exponential density). Cells were briefly harvested with 0.25% trypsin, washed with sterile PBS, counted, then injected in a volume of 200 μl as 2–5 X 10^6^ cells mixed with an equal volume of phenol red-free Matrigel™ into the fourth left mammary fat pad. Controls received sterile PBS mixed with an equal volume of phenol red-free Matrigel.™ Mice were maintained under isofluorane anesthesia (2.5 ml/min) for 5 min/injection. Mice were weighed twice each week and received cell injections once they reached 18 g The fourth mammary fat pad is large enough to be felt through the skin of the BALB/c nude mouse, so injected cells with Matrigel™ were visible as a small bump until tumor growth became palpable. Tumors were measured with calipers twice a week and volumes were calculated using the formula V = (L X W^2^)/2, where L is tumor length and W is tumor width, in millimeters (mm) [19]. Chemotherapy was initiated when tumor volume surpassed 100 mm^3^ and was maintained throughout the study. Doxorubicin (5 mg/kg) and trastuzumab (4 mg/kg) were administered by intraperitoneal injection on successive days each week, while untreated mice received injections of sterile PBS.

### Cell culture

#### Breast cancer cells

Her2^+^ human breast cancer cells (BT474) were purchased from the American Type Culture Collection (ATCC) and grown in culture according to the supplier’s directions. Human BT474 cells were grown in Hybri-Care Medium (ATCC) supplemented with 10% fetal bovine serum, L-glutamine and penicillin-streptomycin on 0.1% gelatin. Cells were passaged at 90% confluence and used in xenografts between passages 3–4.

#### Endothelial cells (EC)

Primary cultures of cardiac endothelial cells were prepared from hearts of 3–5 week-old C57BL/6 or B6NU mice using the protocol previously described for primary endothelial cell isolation from mouse testes [20]. Hearts were removed from euthanized mice and rinsed with sterile PBS to remove residual blood. Hearts were dissected free of great vessels, minced with scissors, and digested with 5 mg/ml type II collagenase (Worthington) for 30–45 min at 37 °C in a shaking incubator at 250 rpm. Collagenase digestion was quenched with an equal volume of FBS. Cells were filtered sequentially through 100 μm and 40 μm filters, washed 3 times with HBSS resuspended in DMEM/F12 supplemented with 10% FBS and pen-strep. Cells were pre-plated for 20 min to remove fibroblasts; non-adherent cells were washed with HBSS, resuspended in EC growth medium (Advanced DMEM supplemented with 15% FBS, EC growth supplement, L-glutamine, and pen-strep) and plated onto 0.1% gelatin-coated dishes overnight. The following day cardiac ECs were harvested by magnetic bead selection with anti-CD31 (Miltenyi Biotech) and expanded on 0.1% gelatin-coated dishes.

### Endothelial cell assays

#### EC viability

Monolayer cultures of EC harvested from murine heart, lung, and liver were assessed for survival after 72 treatment of PBS, 0.1 μM or 1 μM doxorubicin using an MTS Viability Assay (Promega).

#### EC tube formation

Early passage [1, 2] heart or lung ECs were harvested with Accutase™ and plated onto basement membrane extract (BME, Cultrex™ R&D systems) at 5 μl/mm^2^ at a density of 1.8 X 10^3^ cells/mm^2^ for EC tube formation. PBS or DOX (1 μM) was added to EC growth medium at 4 h and tube survival was documented by microscopy.

### Echocardiography

Mice were anesthetized by inhalation 1.5–2% isofluorane via nose cone and placed on a heating pad to maintain body temperature; core temperature was monitored with a rectal probe. Echocardiography was performed using an MS400 probe on a Vevo2100 (FUJIFILM VisualSonics) by the Mouse Cardiovascular Phenotyping Core of the Penn Cardiovascular Institute, Perelman School of Medicine. Data were analyzed with the heart function package provided by VisualSonics Systems.

### Histology

Hearts from study animals were flushed with PBS, fixed with 4% paraformaldehyde overnight, and maintained in 100% ethanol prior to dehydration and paraffin embedding (Molecular Cardiology Service Center, Penn Cardiovascular Institute, Perelman School of Medicine). Sections were stained for Masson’s Trichrome and Picrosirius Red (Direct Red 80, Sigma-Aldrich) using established methods. Images were acquired using an Olympus BX60 microscope equipped with Nikon DS-Fi2 and Qi-MC cameras and Nikon Elements processing software. Images of whole-heart histology were obtained with a Zeiss Axio Imager 2**;** images were motorized scanned and stitched with ZEN imaging software. Representative areas of each montage were used to calculate the percentage of fibrosis using ImageJ (NIH).

### Miles permeability assay

Mice were injected intraperitoneally with 100 μl filter-sterilized Evans Blue (EB) dye (2% in sterile PBS) and allowed to move freely in the cage for 2 h, allowing dye circulation. Following euthanasia, hearts were flushed with PBS and cut in half along the anterior-posterior axis, to expose both ventricles and the interventricular septum. One half was flash-frozen in isopentane chilled in dry ice and the other half was weighed and placed in dimethylformamide to extract the EB. Samples were extracted for a minimum of 48 h at room temperature on a rotating shaker at 100 rpm. Samples were mixed with an equal volume of PBS and read as duplicates using black-walled 96-well plates, with an excitation wavelength of 620 nm and emission wavelength of 680 nm using a Biotek Synergy H1 plate reader. The amount of EB extravasated into the tissue was calculated from the measured fluorescence, relative to an EB standard curve, and expressed as μg EB/g tissue.

### Fluorescence microscopy

Frozen sections of EB-labeled hearts were stained with rat anti-CD31 (BD Biosciences) primary antibody and goat anti-rat Alexa 488 (Invitrogen) secondary to visualize the endothelium of blood vessels. Extravasated EB dye was localized as red-orange fluorescence. Sections from unlabeled (no EB injection) samples served as controls for background fluorescence.

### Enzyme-linked immunosorbent assay (ELISA)

Serum was prepared from whole blood samples taken from mice at the time of euthanasia prior to flushing hearts with PBS. The amount of cardiac troponin-I (cTnI) and brain natriuretic peptide (BNP) in serum was measured with ELISA kits from Life Diagnostics, Inc., and Ray Biotech, respectively, Samples were assayed in duplicate relative to purified mouse cTnI or BNP standard curves and read at 450 nm using a Synergy H1 plate reader (BioTek Instruments, Winooski, VT).

### TUNEL assay

Apoptosis in frozen sections of heart tissue was detected using the Dead-End™ TUNEL System (Promega) to label fragmented DNA with fluorescein-12-dUTP according to the manufacturer’s protocol. Labeled sections and positive controls were observed by fluorescence microscopy (Olympus).

### Protein analysis

Total protein was extracted from samples of hearts, lungs, and gastrocnemius muscles in RIPA buffer (Sigma-Aldrich, St. Louis, MO) and quantified using a Pierce BCA assay (ThermoFisher Scientific, Waltham, MA). Proteins were separated on 10% Bis-Tris NuPAGE gels in MOPS buffer (ThermoFisher) and transferred to Immobilon-P PVDF membranes (Millipore-Sigma). Blots were washed with Tris-buffered saline (TBS)-Tween 20 (10 mMTris, 0.9% NaCl, 0.1% Tween 20, TBS-T), blocked for non-specific binding with 5% nonfat milk in TBST, and incubated with primary antibodies to specific proteins, β-actin (Sigma-Aldrich A2668), ZO-1, occludin, claudin, and JAM-A (Santa Cruz Biotechnology, sc33725, sc133256, sc81796, and sc53624). Blots were washed in TBST and incubated with secondary antibodies (Cell Signaling Technology 7074, 98,164, and 91,196) linked to horseradish peroxidase (HRP). Protein bands were visualized using enhanced chemiluminescence (100 mM Tris pH 8.6, 0.2 mM p-coumaric acid, 1.25 mM luminol) documented with a FluorChemE imager (ProteinSimple, SanJose CA).

### Statistical analysis

Sample means and standard errors were calculated and differences between experimental groups were compared using a Student’s *t-*test (GraphPad Prism 8.0). *p* < 0.05 was considered statistically significant.

## Results

### Breast tumor growth in nude mice was inhibited by DOX/TRZ treatment

**Fig. 1:**
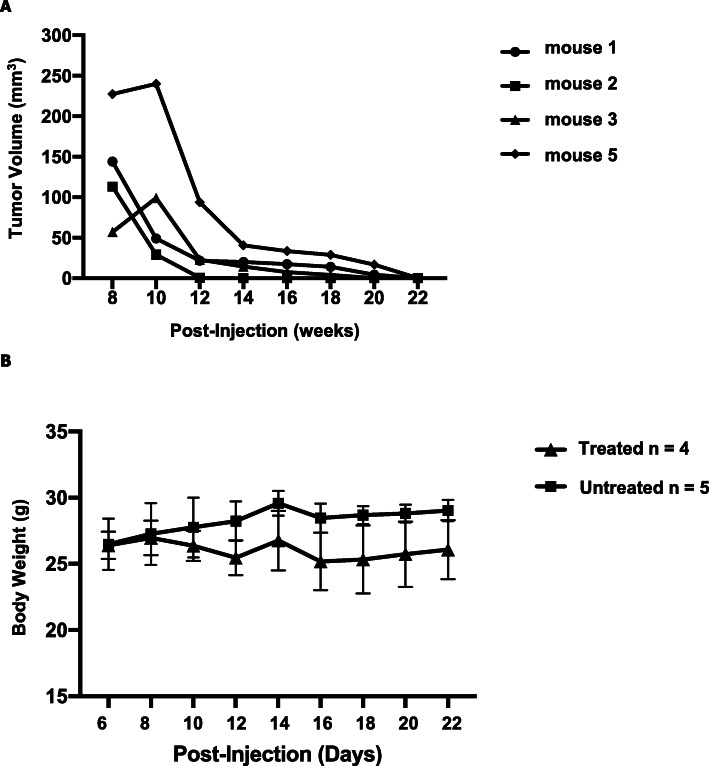
Suppression of breast tumor growth in Nude mice inoculated with Her2+ human breast cancer cells (BT74) after doxorubicin (5 mg/kg/wk) and trastuzumab (4 mg/kg/wk) treatment. **a** Tumor volume measured on indicated days for individual mice after tumor cell inoculation with weekly doxorubicin/trastuzumab treatment initiated when tumor volume reached 100 mm3. **b** Average body weight of tumor-bearing mice treated with 5 mg/kg doxorubicin/4 mg/kg trastuzumab (treated; *n* = 4) and mice without tumors (untreated; *n* = 5) were given PBS. Mice in these experiments followed Study design II (see Table 1 in Methods)

Athymic BALB/c nude mice that were inoculated in the mammary fat pad with HER2+ human breast cancer cells (BT474) developed palpable tumors within four to 8 weeks. When breast tumors reached 100 mm^3^ in size, DOX/TRZ treatment was initiated and tumor growth monitored by calipers. Tumor volumes decreased steadily in response to treatment (Fig. 1a) to minimally palpable or even non-palpable, with no apparent toxicity as indicated by increasing body weight (Fig. 1b).

### Impaired cardiac function after DOX/TRZ treatment in a mouse model of breast cancer

**Fig. 2:**
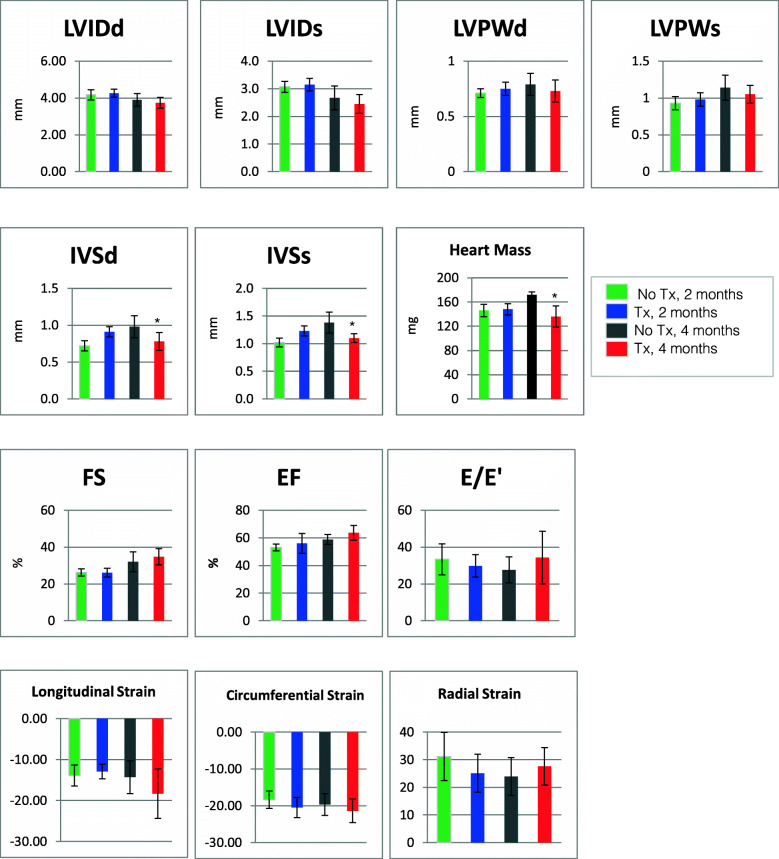
Echocardiography of Treated vs Untreated Hearts. Mice in these studies were treated in study design I and study design II (see Table 1 in Methods section). LVIDd, left ventricular internal diameter end diastole; LVIDs, left ventricular internal diameter end systole; LVPWd, left ventricular posterior wall thickness, end diastole; LVPWs, left ventricular posterior wall thickness, end systole; IVSd, interventricular septal end diastole; IVSs, interventricular septal end systole; Heart Mass, direct measure; FS, fractional shortening; EF, ejection fraction; E/E’, ratio of mitral peak velocity of filling (E) to early diastolic mitral annular velocity (E’); Longitudinal Strain, long axis longitudinal strain (shortening); Radial Strain, long axis radial strain (thickening); Circumferential Strain, short axis circumferential strain (shortening); *p < 0.05

We used echocardiography to assess cardiac function in mice after 2 months and 4 months of DOX/TRZ treatment, compared to control mice that did not receive treatment (Fig. 2). After 2 months of treatment there was no significant change in cardiac function, compared to untreated controls. After 4 months of treatment, the average mass of hearts from treated mice showed a significant decrease (*p* < 0.05) compared to the hearts of untreated controls. Similarly the thickness of the interventricular septum in diastole and systole (IVS_d_, IVS_s_, respectively) showed a decrease after 4 months of treatment (p < 0.05) as compared to the untreated hearts. Cardiac deformation as measured by long axis longitudinal strain (shortening), long axis radial strain (thickening), and short axis circumferential strain (thickening) all showed modest differences but no statistically significant changes. Although there was no significant change in cardiac function after 4 months of DOX/TRZ treatment, the ejection fraction (EF) was maintained, along with a worsening of diastolic function (E/e’). Although there is not definitive proof of heart failure and our data indicate mild diastolic dysfunction and cardiac remodeling which is consistent with subclinical cardiotoxicity, it is possible that this phenotype may be in line with heart failure with preserved ejection fraction (HFpEF) [21].

**Fig. 3:**
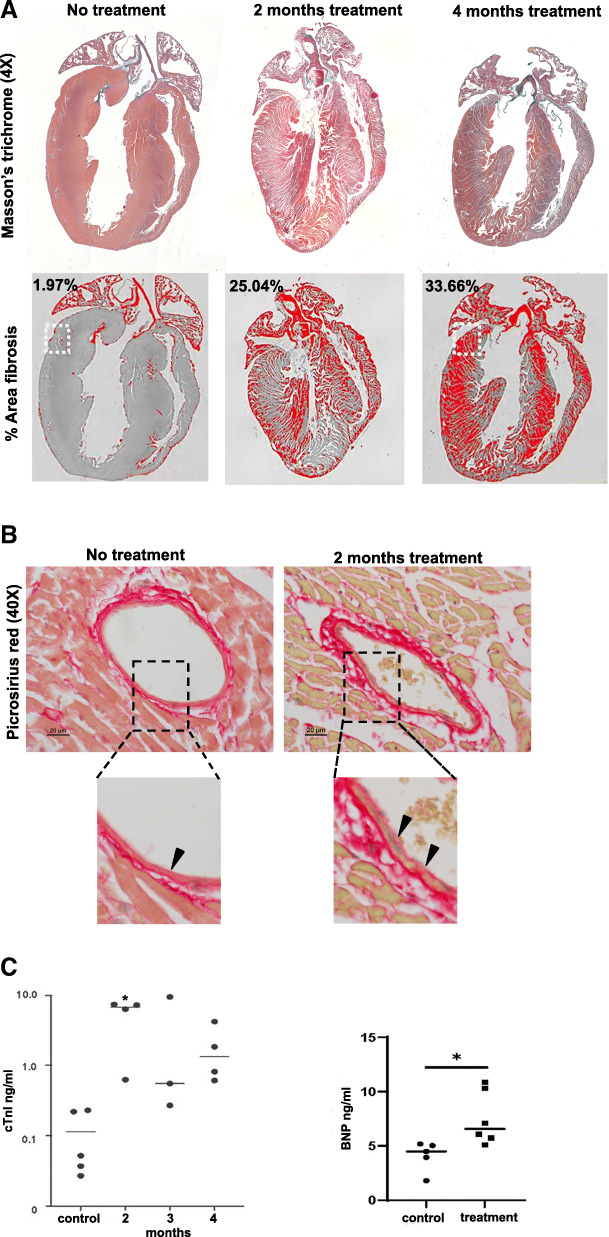
Cardiac damage in hearts from DOX/TRAZ treated tumor-bearing mice compared to untreated mice. **a** Images of heart sections after Masson’s trichrome (top row) staining at the indicated times: no treatment, 2 months treatment or 4 months treatment. Bottom row: Images of heart sections used to calculate percentage of fibrosis (indicated on images) by ImageJ. **b** Picrosirius red staining of heart section after the indicated treatment shows the distribution of collagen fibers in thin layers of connective tissue surrounding cardiomyocytes and blood vessels in untreated heart. Magnified image of no treatment section shows a normal thin layer of connective tissue under the smooth with arrow indicating intact endothelial lining. Magnified image from 2 months of treatment indicate increased collagen in connective tissue layer surrounding blood vessels from treated hearts and a disruption of the endothelial lining of vessels, showing an uneven surface and swollen, necrotic cells (arrows). **c** Quantification of Cardiac troponin I (cTnI) and Brain naturetic protein (BNP) levels by ELISA revealing elevated cTnI levels in serum from DOX/TRAZ treated mice compared to untreated controls (*n* = 5), ** *p* < 0.01, Student’s T-test. Control = 0.113 ng/ml; 2 months treatment = 5.45 ng/ml (*n* = 4); 3 months = 3.46 ng/ml (*n* = 3); 4 months = 1.88 ng/ml (*n* = 4) cTnI. BNP was elevated in serum from DOX/TRZ treated mice after 4 months treatment as compared to untreated controls; control = 4.04 ng/ml BNP (*n* = 5); treated = 7.52 ng/ml (*n* = 6), *p < 0.05, Student’s T-test. Mice in A were treated as described in study design I, mice in B were treated as described in study design II (see Table 1 in Methods)

Gross examination of the heart, lungs, liver and other organs during tissue harvest showed no differences between the treated and untreated tissues (data not shown). However, histological evaluation of heart sections stained with Masson’s trichrome with Alcian blue-stained connective tissue in the heart (Fig. 3a) show a progressive increase in fibrosis revealed by widespread blue staining throughout the walls of the ventricles following two and 4 months of DOX/TRZ treatment. In normal (untreated) cardiac muscle, the connective tissue is visible as thin blue lines in the interstitial spaces around groups of cardiomyocytes and blood vessels, comprising only 1.9% of the stained area. Our data show a striking 25% increase in the amount of blue staining surrounding the cardiac muscle fibers as well as the blood vessels after 2 months of treatment and a further increase to 34% after 4 months of treatment (Fig. 3a). We used picrosirius red staining for collagen (I and II) fibers to elucidate the extent of fibrosis following DOX/TRZ treatment. In untreated hearts, collagen is visible as discrete red lines bordering the cardiac muscle fibers and arranged in layers in around vessels with vessels displaying a smooth endothelial lining. In vessels that have been exposed to DOX/TRZ, there are thicker layers of collagen around the cardiac fibers and striking hypertrophy in the external layers of the vessels, with an uneven endothelial lining of exposed vessels with disruptions (Fig. 3b).

Damage to cardiac muscle in our mouse model of breast cancer following two and four-months of DOX/TRZ treatment was confirmed by increased serum levels of cardiac troponin I (Fig. 3c) as compared to untreated controls. Cardiac troponin I levels were 30-fold higher in treated mice compared to untreated controls. Serum levels of brain natriuretic peptide (BNP), another biomarker of cardiac stress, were 1.9 times higher in treated mice compared to untreated controls (Fig. 3c).

### DOX/TRZ treatment may increase cardiac vascular permeability

**Fig. 4:**
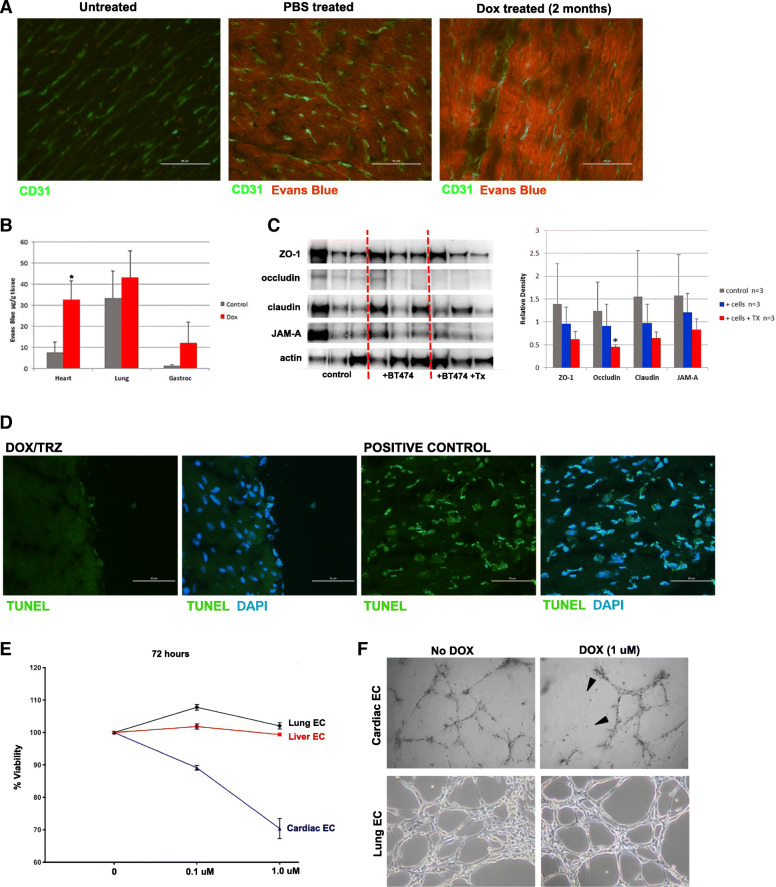
Cardiac endothelial cells (ECs) are sensitive to DOX. **a** Representative images of heart sections immunostained with the EC marker CD31 (green) before or after intraperitoneal injection with Evans Blue (EB) dye to assess vascular permeability with EB dye visible as red fluorescence. **b** miles Assay of EB extracted from labeled tissues shows 4 times more dye in hearts, but not lung or gastrocnemius muscle, from DOX-treated mice, compared to untreated. *p < 0.05. **c** Western blot analysis of junctional proteins in hearts from control mice (no tumor), tumor-bearing mice (+ BT474) or tumor bearing-mice treated with 5 mg/kg DOX and 4 mg/kg TRZ (+BT474 + Tx). Each line is an individual mouse. Graph on right shows quantification of Western blot relative density relative to β-actin and was determined using ImageJ. **d** Images of heart sections treated with DOX/TRZ and stained with TUNEL +/− DAPI or cells treated with DNase I as a positive control for TUNEL expression. **e** Quantification of liver, lung and cardiac endothelial cells (EC) survival in response to doxorubicin treatment at the indicated concentration over 72 h. **f** Images Cardiac and lung ECs grown on basement membrane extract (BME) forming 2-dimenensional tube-like structures in the presence or absence of indicated concentration of doxorubicin (DOX). Mice were treated as described in study design III (see Table 1 in Methods)

To assess the integrity of the cardiac vasculature, we used the Miles assay in which Evans blue dye binds to serum albumin and serves as a marker of blood vessel permeability [22]. Under normal conditions there is minimal leakage of the serum albumin into tissue spaces, however damage to vasculature will lead to increased permeability with Evans blue detected in the tissues. After 2 months of DOX/TRZ treatment in breast tumor bearing-mice, we injected Evans Blue and examined the hearts by immunofluorescence and quantification of Evans blue dye. Immunostaining with the endothelial marker CD31 localized blood vessels in heart sections and since Evans blue dye fluoresces at 680 nm, we examined heart sections in the red fluorescent channel. In the absence of Evans blue there was no detectable signal in the red channel (Fig. 4a) and only a background level of red fluorescence visible in heart sections from control mice that were injected with Evans blue (Fig. 4a). In contrast, mice that received DOX/TRZ treatment for 2 months displayed a strong red fluorescent signal in heart sections compared to controls (Fig. 4a). For the Miles assay, we extracted the Evans blue dye from heart, lung, and gastrocnemius muscle samples and quantified levels spectrophotometrically. Tumor bearing mice treated with DOX/TRZ showed four times more Evans blue dye extravasation into heart tissue as compared to untreated mice. Lung and gastrocnemius also showed an increase in dye content following DOX/TRZ treatment, but not at the level of significance seen in heart (Fig. 4b).

### DOX/TRZ treatment may involve changes in junctional proteins

Heart lysates from control mice, mice inoculated with BT474 breast cancer cells or mice inoculated with BT474 cancer cells and treated with DOX/TRZ treatment were probed for tight junction-associated protein ZO-1, occludin, claudin, and JAM-A by Western blot with expression levels of each protein quantified relative to β-actin (Fig. 4c). Although all of the junctional proteins showed a decrease following DOX/TRZ treatment, only the decrease in occluding expression was found to be statistically significant (Fig. 4c**)**. Interestingly, there was a decrease in the expression of junctional proteins in tumor-bearing mice in the absence of DOX/TRZ treatment further supporting the notion that breast tumors and the presence of cancer cells may effect cardiac vasculature. We found minimal evidence of overall cell death in hearts of tumor-bearing mice treated with DOX/TRZ as detected by TUNEL assays and in comparison to tumor cells treated with DNaseI as a positive control for TUNEL staining (Fig. 4d).

### Cardiac endothelial cell viability and function are impaired after DOX treatment

It has become increasingly evident that the endothelium functions in an organ specific manner to regulate developmental processes, maintain normal organ homeostasis, and control the response to vascular injury and inflammation. Published studies revealed differential gene expression by cardiac ECs versus other organ ECs such as the brain, kidney or lung [23]. To investigate whether cardiac ECs were more sensitive to DOX, we isolated and cultured primary ECs from the heart, lung and liver of wild-type mice and exposed them to 0.1 μM and 1 μM doxorubicin for 72 h. EC viability was measured and show that cardiac ECs treated with low dose DOX (0.1 μM) doxorubicin had 10% decrease in viability that decreased even further by 30% when treated with a moderate dose (1 μM) of DOX. In contrast to cardiac EC, lung and liver EC did not show significant cell death at any DOX dose (Fig. 4e).

One measure of EC function is the ability to form two-dimensional (2D) tube-like structures on Matrigel, a basement membrane extract preparation. When ECs are seeded at high density, they readily form connected 2D tube-like structures (Fig. 4f). Upon exposure to 1 μM DOX for 6 h, cardiac EC tubes began to break down, losing their connections and tube-like structures disappear. In contrast, 2D-tubes generated by lung ECs maintained their connections in the presence of 1 μM DOX after 6 h (Fig. 4f).

## Discussion

We examined cardiotoxicity in response to combined DOX/TRZ therapy in an orthotopic mouse model of breast cancer. The growth of Her2-positive human breast cancer cells inoculated into the mammary fat pad of Nude mice was suppressed by treatment with DOX/TRZ. Using clinically relevant doses of DOX/TRZ, tumor bearing-mice were treated for two to 4 months, with mice continuously gaining weight indicating the lack of cachexia often seen when the dose of drug is toxic. Combination drug treatment in our mouse studies utilized an alternating schedule (DOX and TRAXZ delivered on separate days in the same week) rather than sequentially as is delivered to patients. This treatment regimen was utilized to both target tumor growth as well as to minimize toxicity. The ability to successfully control tumor growth and the lack of whole-body toxicity suggests that this is as an effective model for cardiotoxicity studies and is a reasonable model for treatment of breast cancer in patients [24–26].

At the two study endpoints, 2 months and 4 months of treatment, cardiac function was assessed by echocardiography (ECHO). ECHOs showed impaired diastolic function as decreased LV filling with elevated LA pressure and a preserved ejection fraction in the treated mice as compared to controls, indicating subclinical cardiotoxicity and a potential profile that may lead to heart failure with preserved ejection fraction [21, 27]. Cardiac mass showed a significant decrease (*p* < 0.05) following 4 months of treatment, and echocardiographic metrics also showed that after the 4 month treatment, there was a significant (p < 0.05) decrease in the ventricular septum thickness in treated compared to untreated mice. Recent clinical studies have indicated that reduced myocardial capillary density (microvascular rarefaction) with reduced coronary flow reserve (maximum increase in blood flow through the coronary arteries above the normal resting volume) contributes substantially to diastolic dysfunction in HFpEF patients [28, 29]. The observation of subclinical cardiotoxicity in DOX/TRZ treated mice is consistent with the observation that cardiac ECs, in addition to cardiomyocytes became damaged during the combination treatment. Cardiac damage was also confirmed by the elevated cTnI levels. Although individual mice within each treatment trial (2 or 4 months) varied with the amount of cTnI measured, the overall trend indicated that treated mice had higher levels of cTnI and BNP, signs of cardiac damage.

Histologic examination of the hearts revealed progressive fibrosis in the ventricular walls, detected by Masson’s trichrome staining following 2 to 4 months of DOX/TRZ treatment, compared to hearts from mice that did not receive treatment. A baseline level of 1.9% connective tissue staining increased to 25% fibrosis after 2 months and 33.6% after 4 months. The increased fibrosis was not accompanied by significant loss of cardiac myofibers, but the accumulation of connective tissue surrounding those myofibers could contribute to decreased flexibility and subsequent compromised relaxation. The picrosirius red staining highlights the thicker layers of collagen surrounding cardiac myofibers in the hearts from treated mice with increased collagen especially apparent surrounding the cardiac vessels, supporting the idea that leakage through the endothelial layer provoked activation of cardiac fibroblasts. The innermost layer of endothelial cells lining the vessels typically appear smooth and continuous, as they appear in the untreated hearts but in DOX/TRZ treated hearts the endothelial layer appears rough with a marked enhancement of collagen immediately below the endothelial lining. Although other studies have demonstrated loss of ECs in DOX-treated hearts via cell counting or a reduction in the number of microvessels detected via immunofluorescent staining, our results imply that cardiac vasculature is damaged leading to leaky cardiac vasculature and not cardiac EC death. We proposed that the impaired gatekeeper function of the endothelial cells comprising the lining of the vessels supplying the myocardium plays a key role in DOX-induced adverse cardiac events.

We demonstrate increased cardiac vascular permeability using the Miles assay, in which the Evans blue dye binds to serum albumin that usually has minimal leakage into the extracellular fluid. When extracted from labeled tissues and quantified, the amount of fluorescence from DOX/TRZ treated tissues was fourfold higher than background fluorescence from untreated controls. The leakage of Evans blue dye into treated hearts was visible as enhanced red fluorescence seen in frozen sections compared to background levels in untreated hearts and unlabeled controls. Hearts appeared more affected by leakage compared to other tissues examined, lung and gastrocnemius muscle. A previous study suggested that DOX/TRZ treatment affects proteins forming the junctional complexes that are necessary for a tight endothelial barrier and that DOX/TRZ may alters its permeability. This study found that DOX/TRZ treatment decreased levels of the tight junction protein ZO-1 in cardiac ECs, but not in dermal ECs or brain ECs grown in vitro [30]. We evaluated protein expression of the junctional proteins ZO-1, occludin, claudin, and JAM-A from hearts harvested from mice inoculated with BT474 breast cancer cells with or without DOX/TRZ treatment and found similar decreases in junctional protein expression as compared to control mice in the absence of cancer cells or the absence of DOX/TRZ treatment. Our data also show reduced expression of these junctional proteins in hearts from mice injected with cancer cells only, but not as significantly downregulated as compared to hearts from mice treated with DOX/TRZ suggesting that the presence of cancer cells may contribute to changes in cardiovascular permeability.

The tissue-specific responses of the cultured ECs, cardiac vs dermal vs brain [30], suggest that cardiac EC may have inherent susceptibility to DOX/TRZ treatment that predispose them to damage. We compared monolayer cultures of ECs harvested from heart, lung, and liver treated with 0.1 μM and 1.0 μM DOX for 72 h and found that only cardiac ECs suffered significant mortality (30%). The cardiac ECs demonstrated similar susceptibility to DOX when grown as 2-D tube as the cardiac EC tubes fell apart 6 h after DOX treatment as compared to lung ECs which maintained their 2-D tube structures in the presence of DOX.

In this study we utilize a mouse model of breast cancer that lacks a functional immune system which offers some caveats for interpretation of our data as the importance of inflammation and immune cell infiltration in chemotherapy-induced cardiotoxicity is not addressed. However, we propose that our model of cancer therapy-induced cardiotoxicity in nude mice provides a tractable and effective model for examining the role of cardiac ECs as the gatekeepers to underlying tissue and organ integrity. Identifying signs of cardiac EC damage that leads to their increased permeability can also provide early biomarkers in advance of irreparable cardiac damage.

## Conclusions

Our studies utilized an orthotopic mouse model of Her2^+^ breast cancer treated with DOX and TRZ. We found that cancer treatment suppressed tumor growth and resulted in a cardiac phenotype characterized by preserved LV ejection fraction, decreased LV mass, and modest elevation of E/e’, consistent with diastolic dysfunction and subclinical cardiotoxicity, which could progress to true heart failure with preserved ejection fraction upon longer exposure to treatment. Our data indicate that cardiac endothelium is more sensitive to DOX/TRZ treatment as compared to other organ endothelium, leading to leaky cardiac vasculature. Future studies should investigate the effect of DOX and TRZ independently to determine the contribution of TRZ towards cardiovascular leakiness in comparison to DOX-induced defects in cardiac vasculature to ultimately test cardioprotective interventions in breast cancer treatment-induced cardiotoxicity.

## Data Availability

The datasets used and/or analysed during the current study are included in this published article and materials are available from the corresponding author upon request.

## Abbreviations

BNP: Brain natriuretic peptide
CTC: Cancer treatment-induced cardiotoxicity
cTnI: Cardiac troponin-I
CV: Cardiovascular disease
DOX: Doxorubicin
EC: Endothelial cells
EF: Ejection fraction
E/E’: Ratio of mitral peak velocity of filling (E) to early diastolic mitral annular velocity (E’)
HFpEF: Heart failure with preserved ejection fraction
IP: Intra-peritoneal
IVS: Interventricular septum
LVID: Left ventricular internal diameter
PBS: Phophate buffered saline
TRZ: Trastuzumab
